# Multidose intramuscular allogeneic adipose stem cells decrease the severity of canine atopic dermatitis: A pilot study

**DOI:** 10.14202/vetworld.2019.1747-1754

**Published:** 2019-11-08

**Authors:** Nathaly Enciso, José Amiel, John Pando, Javier Enciso

**Affiliations:** 1 Laboratorio de Cultivo Celular e Inmunología, Universidad Científica del Sur, Lima, Perú; 2 Departamento de Bioquímica y Biología Molecular, Facultad de Veterinaria, Universidad Complutense de Madrid, Madrid, Spain; 3 Department of Cytometry, Institute of Cell Therapy. CRIOCORD. Lima. Peru

**Keywords:** adipose stem cells, allogeneic stem cells, canine atopic dermatitis, cellular therapy

## Abstract

**Aim:**

The aim of this pilot study was to evaluate the therapeutic and safety performance of an intramuscular treatment protocol of multidose of allogeneic adipose stem cells (ASCs) isolated, characterized, and expanded *ex vivo* from a healthy canine donor.

**Materials and Methods:**

Twelve dogs diagnosed with canine atopic dermatitis (CAD) were intramuscularly treated with 0.5×10^6^ of cryopreserved ASCs from a healthy immunized young canine *Ehrlichia canis* free donor weekly for 6 weeks. Treatment efficacy was evaluated by the pruritus index and the CAD Lesion Index (CADLI) test. Safety and adverse effects were determined by injection site reaction, weight, blood chemistry, liver function, and whole blood count.

**Results:**

Canine ASCs obtained from a donor met the minimum qualities required for this type of cells and showed viability of 90% after thawing. The efficacy of the CADLI score and the pruritus index in 12 dogs with atopic dermatitis was statistically significant efficacy. No adverse reactions were observed at the intramuscular application site, or in relation to animal weight, blood cell populations, or liver and renal function.

**Conclusion:**

These results suggest that intramuscular administration of cryopreserved ASCs to dogs with atopic dermatitis is a promising cellular therapeutic product for the relief of the symptoms of this disease; however, the duration of the effects obtained with this dose and with other doses should be evaluated, as well as possible immune reactions. As far as we know, this is the first report of the use of multiple intramuscular doses cryopreserved ASCs to treat atopic dermatitis.

## Introduction

Canine atopic dermatitis (CAD) is a common, genetically predisposed inflammatory and pruritic allergic skin disease with characteristic clinical features [1,2], having a prevalence of 20-30% [3]. In terms of origin, this disease is complex and multifactorial and involves genetic factors [4,5], immune dysregulation, allergic sensitization, skin barrier defects, cutaneous microbiome, and environmental factors [6-8]. The pathogenesis of canine and human atopic dermatitis is not fully understood [9]. In humans, some authors consider it to be a systemic Th2-dependent disease [10], while for others, it is primarily a chronic skin inflammation [11]. Similarly, CAD is considered as an inflammatory disease associated with an aberrant Th2 pattern immune reaction [12]. For this reason, the criteria for diagnosis are based on signs established in clinically recognized guidelines [13]. Given the complex and multifactorial nature of CAD, treatment differs in overtime and geographically. Several randomized controlled clinical trials, as well as systematic reviews, have proposed a choice of prospective drugs that can benefit patients with CAD [14-16], who are also often treated with allergy immunotherapy [17].

Recent clinical studies have shown that CAD treatment has mainly been based on the concept of T-cell immune dysregulation as a major immunological deficiency in atopic dermatitis. Therefore, the current treatment includes agents involving cytokine modulation and/or growth factor receptor expression [18,19]. Taking this into account, it has been proposed that blocking chemokine receptor antagonists and/or drugs involved in the inhibition of T-cell activation might have a beneficial effect on CAD [20,21], although short- and long-term side effects still remain an issue [22,23]. On the other hand, the use of mesenchymal stem cell (MSC) therapy, especially bone marrow-derived MSCs (BM-MSCs) and adipose stem cells (ASCs), has mainly been supported by the particular traits of these cells, including cell renewal, differentiation, and production/release of a vast array of growth factors, cytokines, and other types of mediators of cell function [24,25]. Furthermore, the use of allogeneic MSC has shown to be effective and safe in canine diseases [26]. In addition, adult MSCs exhibit attractive immunomodulatory properties in degenerative diseases in animals [27]. This is the case of ASCs, a type of MSC, which in addition to the cellular and molecular traits of BM-MSC, exhibits robust immunosuppressive activity, mediated by induction of the production of Tregs [28] and the selective release of several types of growth factors [29-33].

Therefore, according to the cellular, molecular, and immunomodulatory properties of ASCs, we aimed to evaluate the therapeutic and safety performance of an intramuscular treatment protocol of multidose of allogeneic ASCs in the treatment of atopic canine dermatitis.

## Materials and Methods

### Ethical approval and Informed consent

Licensed veterinary clinicians conducted all the animal procedures. The protocols were approved by the Universidad Científica del Sur’s Ethics Committee from Lima, Peru (approval reference number 004-2013), and written informed consent was obtained from the owners.

### Patients

Twelve dogs with a clinical diagnosis of atopic dermatitis were enrolled in the study for 6 weeks. The patients were between 1 and 3 years of age and were determined to be healthy on physical examination. Before the study, routine serum chemistry and hematology evaluations were performed to ensure overall systemic health. The diagnosis of atopic dermatitis was based on the clinical history, compatible clinical signs, and exclusion of other pruritic skin disease and met the criteria established by Favrot *et al*. [13]. The patients had not received antihistamines, glucocorticoids, and cyclosporine treatment during 2, 4, and 8 weeks, respectively, before the study.

### Characteristics of the cell product for infusion

#### Isolation of ASCs

Considering the efficiency in obtaining high cell counts and minimal induced morbidity during cell collection [34], it was decided to use the omentum adipose tissue as a source of ASCs.

Tissue sample (5 g) from omental fat deposits was obtained from a donor dog, clinically healthy female, mongrel, 1 year old, 8 kg of weight that met the full annual immunization program, including canine distemper, canine parvovirus, parainfluenza, adenovirus type 2, canine coronavirus, Leptospira, and being Anaplasma, Dirofilaria, Lyme, and *Ehrlichia canis* free using a SNAP 4Dx Plus test (Idexx, Westbrook, United States). In addition, a chromatographic test was performed previously to rule out the presence of *E. canis* in blood. The fat deposits were obtained using a standard surgical procedure under anesthesia with intravenous xylazine/ketamine (1/10 mg/Kg body weight). The donor’s owner provided informed consent allowing the use of extracted tissue for research purposes.

The samples were placed in conical centrifuge tubes (FALCON, Thermo Fisher Scientific, Waltham, Massachusetts, USA) containing 25 mL of Dulbecco’s Modified Eagle’s Medium (DMEM) (Sigma-Aldrich, St. Louis MO, USA) containing 100 U/ml penicillin/streptomycin/amphotericin (Millipore, Merck KGaA, Darmstadt, Germany) and sent to the Good Manufacturing Practice facility at the university for processing.

Adipose tissue samples were washed with phosphate buffer solution (PBS) to eliminate blood clots and were then cut and incubated in a PBS containing 1 mg/ml (Sigma-Aldrich, St. Louis MO, USA) of collagenase type I and 1% of antibiotic (penicillin, streptomycin, and amphotericin, Millipore) for 1 h at 37°C with slight continuous agitation in a shaker. At the end of the incubation period, a solution containing 10% fetal bovine serum (FBS; Gibco, Thermo Fisher Scientific, Waltham, Massachusetts, USA) in DMEM was added to stop the collagenase reaction. Afterward, it was filtrated through a cell strainer of 100 μm and was centrifuged in at 800 RPM (Boeco C28A, Germany) for 5 min. The cell pellet, containing few native ASCs, was saved and transferred in culture flasks (Nunc, 75 cm^2^) with DMEM, 10% FBS and 1% penicillin-streptomycin (Sigma-Aldrich, St. Louis MO, USA).

The cells were cultured in a humidified incubator at 37°C at 5% CO_2_. After 3 days, the wells were washed 3-4 times with PBS and the medium was recharged. Cells in the developing adherent layer were cultured for 9-12 days, changing the culture medium every 3 days. After reaching 80% of cell confluence in the developing adherent layer (primary ASCs), the cells were detached using Trypsin-EDTA (0.05% Trypsin-0.5 mM EDTA; Sigma-Aldrich, St. Louis MO, USA) for 3 min, neutralized with the same volume of DMEM medium with 10% (FBS, Gibco, Thermo Fisher Scientific), and centrifuged at 800 rpm for 5 min. The cells were expanded by successive passages, up to passage 5.

When the targeted number of expanded ASCs was attained, the cells were detached from the culture vessel, thoroughly washed and suspended in DMEM. Aliquots were taken for cell counting, viability assay, microbiological testing, and MSC characterization. The expression of pluripotency factor Oct4 was detected by immunocytochemistry using a monoclonal antibody (Invitrogen).

### Flow cytometry immunophenotyping

Phenotypic identification of the ASCs was performed with the use of conjugated monoclonal antibodies: Anti-canine CD90PE (VMRD, USA), anti-canine CD 105FITC (VMRD, USA), and anti-canine CD45PECy7 (VMRD, USA). Three hundred microliters of ASCs suspension containing 200,000 cells were placed in Eppendorf tubes and incubated for 30 min at 4°C with 15 μL of monoclonal antibodies. Afterward, 500 µL assay buffer (Millipore) and 300 µL of flow count (Beckman Coulter) were added. A minimum of 10,000 events was acquired and analyzed with a Flujo Guava® easyCyte flow cytometer (Merck), and cytometry data were analyzed using Guava® InCyte™ software (Merck).

### Differentiation

The multipotentiality of MSCs was determined by establishing the differentiation capacity in three lineages. For this purpose, ASCs (5×10^3^/cm^2^) from the 5^th^ passage were cultured with DMEM-F12 medium with 10% FBS and 1% antibiotics (penicillin/streptomycin/amphotericin B) for 48 h at 37°C, 5% CO_2_, and 95% humidity in 12-well cell culture plates containing covers treated with poly-L-lysine 0.1% and sterilized by ultraviolet exposure for 12 h.

### Osteogenic differentiation

Osteogenesis differentiation medium (StemPro, Gibco) was used as per the manufacturer’s instructions for osteogenic induction. ASCs were cultured for 21 days, the medium was changed every 3^rd^ day, and differentiation was assessed by Alizarin Red S (Sigma-Aldrich) staining, in which the cells were fixed with 4% formaldehyde solution for 30 min followed by rinsing with distilled water and staining with 2% Alizarin Red S solution (pH 4.2) for 2-3 min. Finally, the cells were rinsed with distilled water and visualized under a light microscope. Red staining indicates the deposition of bone mineral (calcium phosphate) by osteoblasts.

### Chondrogenic differentiation

Chondrogenesis differentiation medium (StemPro, Gibco) was used as per the manufacturer’s instructions for chondrogenic induction. ASCs were cultured for 14 days, the medium was changed every 3^rd^ day, and differentiation was assessed by Alcian blue staining. The cells were fixed with 4% formaldehyde for 30 min and then washed with PBS followed by the addition of 1% Alcian blue solution prepared in 0.1 N HCL for 30 min. Finally, the ASCs were washed with 0.1 N HCL and distilled water was added. Blue staining indicates chondrocyte synthesis of proteoglycans. As a negative control, an equal number of cells were maintained in micromass culture supplemented with an expansion medium for up to 14 days.

### Adipogenic differentiation

An adipogenesis differentiation kit (StemPro, Gibco) was used according to the manufacturer’s instructions for adipogenic induction of the ASCs. The ASCs were cultured for 21 days; the medium was changed every 3^rd^ day, and differentiation was assessed by the presence of lipid droplets that were recorded after staining with Oil Red O stain. Red staining indicates the presence of lipids. As a negative control, an equal number of cells were maintained in the expansion medium for 21 days.

### Cryopreservation of ASCs

Due to the design of the study (6 successive weekly doses), ASCs for injections underwent short-term (up to 10 days) cryopreservation on liquid nitrogen. Cryopreservation was performed by adding a cryopreservation solution (DMEM containing 10% FBS and 5% dimethyl sulfoxide) at a dose of 5×10^6^/cryovial. The resulting cell suspension was cryopreserved until the day of application to the patient, at which time the cells were rapidly thawed according to a previously described protocol [35] and suspended in 2 mL of physiological serum to be injected immediately. At this time, an aliquot was obtained to evaluate the number of cells, viability by means of the blue trypan solution which should be equal to or >90% and microbiological culture.

### Injection of the cell product

Following our routine practices for skin preparation and intramuscular injection in laboratory animals, 0.5×10^6^ cells/kg were intramuscularly injected weekly for 6 weeks into the dog’s middle buttock. At the end of the treatment protocol (6^th^ week), each dog had received a total of 3.0×10^6^ allogeneic ASCs cells/kg at room temperature (20-22°C).

### Study type, design, and patient population

**Figure-1:**
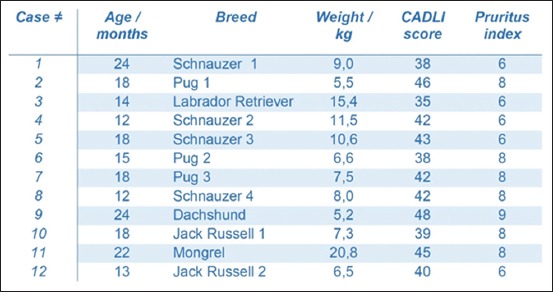
The clinical characteristics of the patients at baseline.

The clinical characteristics of the patients at baseline are shown in Figure-1. Feasibility in this work referred to the proportion of cells that survive cryo-freezing, after expanding the allogeneic ASCs *ex vivo* to reach a sufficient number of cells to obtain the multiple doses necessary for each patient after thawing, for 6 consecutive weeks.

To evaluate whether a stem cell treatment is safe, patients were monitored by both, physical examinations and laboratory tests, after treatment and during the study period. The absence of adverse events, graded according to the Veterinary cooperative oncology group-common terminology criteria for adverse events (VCOG-CTCAE) version 1.0 [36], was considered safety outcomes. An assessment of (1) local inflammation (heat, swelling, and function deterioration) in addition to the presence of new ulcers and worsening of existing ones; (2) systemic damage; and (3) any serious adverse events, leading to hospitalization or death was undertaken. At the same time, the clinical evidence of a decrease and/or absence of the most relevant initial characteristics related to atopic dermatitis and/or its comorbid conditions, recognized through the corresponding indexes were considered efficacies.

The dogs were evaluated for clinical lesions using the CAD Lesion Index (CADLI) and the pruritus index to record the extent and severity of lesions immediately before the pre-treatment and at 6 weeks of post-treatment.

The CADLI is a validated tool for the assessment of indicated body regions, integrating the severity and extent of the lesion(s) in a determined area. Scores from 0 (normal) to 5 (most severe) were obtained, yielding a final score of 0-50 based on the criteria established by plant [37]. To assess pruritis, the dog owners used the pruritus index which is a visual analog scale ranging from 0 (no pruritus) to 10 (extremely severe pruritus) including features of severity and behavior [38].

### Blood chemistry and whole blood count

Blood chemistry tests were performed to determine the levels of the following analytes: Glutamic oxaloacetic transaminase, glutamic pyruvic transaminase, alkaline phosphatase, urea, and creatinine using a biochemical analyzer (SINOWA, BS3100). The whole blood count was performed in a hematologic analyzer (Rayto RT-7600) at day 0 and at the end of treatment.

### Statistical analysis

Data were expressed as the mean±standard deviation and the analyses were carried out using software programs (GraphPad Software, Inc., San Diego, California. United States). Shapiro-Wilk tests were used for testing the normality of data. The analysis of data was performed using the Wilcoxon test. Results were considered statistically significant when p<0.05.

## Results

### Characteristics of the cell product for injection

**Figure-2:**
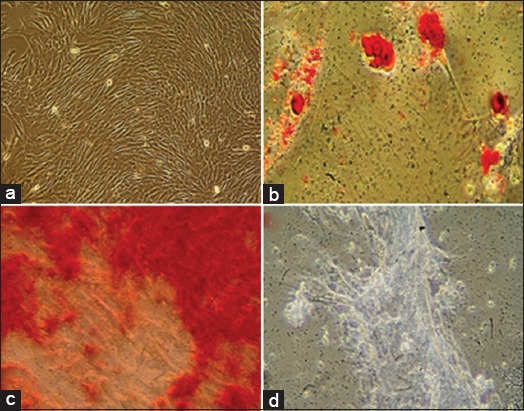
(a) Adipose tissue-derived canine mesenchymal stem cells (MSCs) from the 4^th^ passage showing a spindle-shaped fibroblastic cell morphology. (b) After 3 weeks, adipose MSCs differentiated according to the conditioning medium added. Cells were stained with Oil Red O staining for adipogenic. (c) Von Kossa staining for osteogenic. (d) Alcian blue staining for chondrogenic differentiation, 40×.

ASCs display a spindle-shaped morphology and a strong attachment to the surface of the culture vessel (Figure-2a). Population doubling time was 32 h, which persisted during the whole expansion procedure.

**Figure-3:**
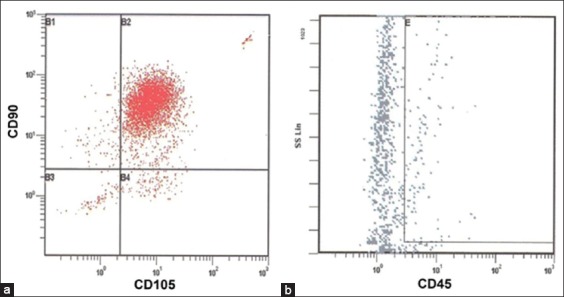
Flow cytometry of the adipose mesenchymal stem cell (MSC) population. Dot plots of adipose MSCs revealed (a) positive results for CD90 (94%) and CD105 (92%) and (b) were negative for CD45 (0%).

Under proper stimuli, *ex vivo* expanded ASCs differentiated from the adipose (Figure-2b), osteo (Figure-2c), and cartilage (Figure-2d) linages. ASCs showed a homogeneous population of cells with high positive CD90 (94%) and CD105 (92%) expression and very low negative CD45 (0%; Figure-3) expression and antigen Oct-4 (>90%) expression by immunocytochemistry, the latter being a transcription factor critical for MSC pluripotency [39]. In addition, immediate post-thaw cell viability was 90%.

### Procedural safety after cell injection

There were no adverse events, clinically significant changes in the vitality signs or physical examination in relation to IM administration of allogeneic ASCs. No significant changes in laboratory test results were observed during the evaluation period. However, there was an increase in body weight at the end of the study period.

### Clinical assessment after ASCs injection

**Figure-4:**
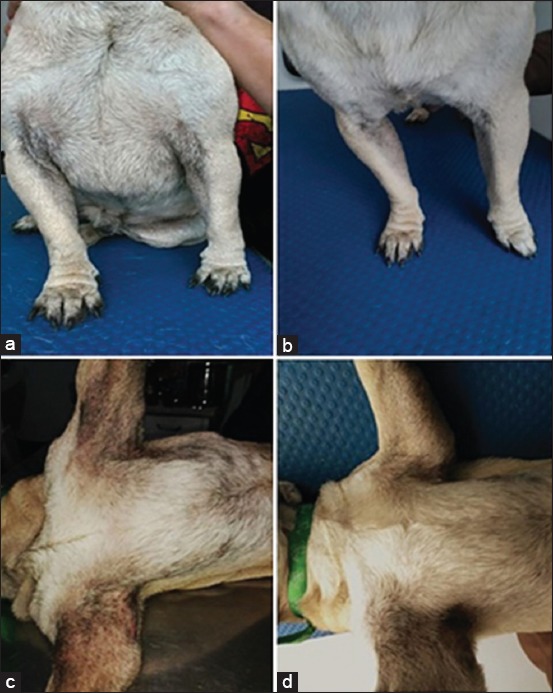
Case 2 (a and b), case 3 (c and d). a and c are pre-treatment and b and d were post-treatment.

Compared to baseline, it was found that the injection of the allogeneic cell product had eliminated and/or diminished most of the symptoms of the disease as well as associated comorbidities prevailing at inclusion, at 6 weeks after the initiation of the stem cell therapy (Figure-4).

**Figure-5:**
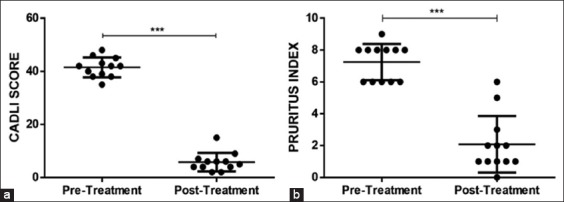
Effect of six doses of allogeneic stem cell therapy in dogs with atopic dermatitis according to the Canine Atopic Dermatitis Lesion Index score [37] and pruritus index [38]. Data represent mean±standard deviation. *** indicates a statistically significant difference of p<0.001.

**Figure-6:**
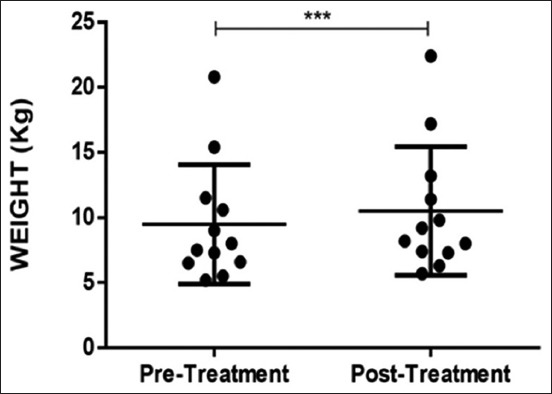
Weight of pre- and post-cellular therapy patients. Data represent mean±standard deviation. ***indicates a statistically significant difference of p<0.001.

In addition, CADLI scores and pruritus index obtained analyses showing statistically significant improvement post-therapy (Figure-5). The mean pre-treatment CADLI score was 41.50±3.729, while the mean post-treatment score was 5.833±3.512. This post-treatment improvement was statistically significant (p<0.001). The mean pre-treatment value of pruritus index was 7.250±1.138, and the post-treatment value was 2.083±1.782, showing a statistically significant improvement post-therapy (p<0.001). The mean pre-treatment value of weight was 9.492±4.582, and the post-treatment value was 10.51±4.944 showing a statistically significant improvement post-therapy (p<0.001) (Figure-6).

## Discussion

In the present study, the intramuscular injection of six doses (0.5×10^6^) of cryopreserved allogeneic ASCs proved to be safe and effective in the treatment of 12 dogs with atopic dermatitis. In all patients, a substantial reduction in pruritus and associated clinical symptoms of atopic dermatitis was observed shortly after injection. This effect of ASCs can be attributed to their previously described qualities including the modulation of Th1/Th2 response, which regulates the pro-inflammatory and pro-pruritic cytokines activated in acute atopic dermatitis [16,40,41]. These beneficial effects can also be related to other mechanisms of stem cell action such as inhibition of effector T-cell proliferation and the expression of inflammatory cytokines by expansion of regulatory T-cells and the production of interleukin-10 [42,43]. Moreover, the action of soluble ASCs factors [44-46] and/or the secretion of paracrine growth factors that can correct several pathophysiological alterations cannot be ruled out [47].

On review of the literature, we found no study on the use of the intramuscular route to apply stem cell therapy in dogs with atopic dermatitis. In addition, it is important to note that IM pathway is safe and minimally invasive alternative to conventional intravenous infusion; it also helps the prolonged survival of transplanted stem cells at the implant site, being a useful alternative to achieve clinical benefits [36,48,49].

These results differ from a study in which allogeneic ASCs were intravenously injected at doses of 1.3 million cells/kg without diminishing the clinical signs of the disease [2]. However, another study carried out in 26 dogs with atopic dermatitis using an intravenous dose of 1.5×10^6^/kg of live weight, achieved a reduction in the clinical lesions without adverse effects [50]. Unlike the previously described studies, in the present study, we report a protocol of six low intramuscular doses of 0.5×10^6^ of cryopreserved ASCs. The low doses reduce the risk of adverse effects induced by the intravenous administration of ASCs and cryopreservation diminishes the cost of production and facilitates immediate use after the diagnosis of atopic dermatitis. Indeed, some studies in animals and humans have shown that high doses of stem cells do not necessarily lead to better efficacy and have even suggested that they may have an inverse dose-response effect. Cell crowding is believed to play a role in this event [51].

It is of note that most of the dog owners recognized that they were grateful for the significant clinical improvements observed following the study after having been asked to report their satisfaction with the treatment. Although this study was not designed to evaluate the dose-response relationship, the clinical outcome revealed that the protocol used for the administration of the cell product (a similar weekly dose of ASCs for 6 weeks) was adequate to decrease skin lesions and itching. Other studies using different drugs for the treatment of atopic dermatitis have suggested that due to the multimodal nature of this pathology, it is necessary to establish the frequency and duration of treatment for each particular drug [52,53].

The results of this clinical study show that the injection of allogeneic ASCs seems to be an effective treatment and a safe clinical approach for the treatment of CAD. These results are in contrast to those of other therapeutic approaches, which in many cases produce acute episodes of immunosuppression associated with systemic infections [54,55], urinary incontinence and lethargy [23], and/or a high risk of developing Cushing syndrome [22]. These results also suggest that trophic factors related to ASCs have regenerative properties.

## Conclusion

The administration of multiple intramuscular doses of cryopreserved allogeneic ASCs to dogs with atopic dermatitis is a promising cellular therapeutic product for the relief of the symptoms of this disease. Nonetheless, the duration of the effects obtained with this dose should be compared with that of other doses, and the mechanism of action and possible long-term immunological reactions should be evaluated. As far as we know, this is the first report on the use of multiple intramuscular doses of cryopreserved allogeneic ASCs to treat atopic dermatitis.

## Authors’ Contributions

JE designed and performed the study. NE contributed in collecting data, interpretation of data, and writing the manuscript. JP and JA participated in collecting data and interpretation of data. JE write and revised the manuscripts. All authors read and approved the final manuscript.
